# Iron metabolism contributes to virulence by enhancing serum resistance in the emerging high-risk clone ST15 CRKP

**DOI:** 10.1080/21505594.2025.2569700

**Published:** 2025-10-07

**Authors:** Jingjing Chen, Hailong Li, Fushun Li, Mingming Kang, Ruixuan Wang, Yunzhuo Chu, Xiaoxu Han, Hong Shang

**Affiliations:** a National Clinical Research Center for Laboratory Medicine, Department of Laboratory Medicine, The First Hospital of China Medical University, Shenyang, China; b Research Unit of Medical Laboratory, Chinese Academy of Medical Sciences, Shenyang, China; c NHC Key Laboratory of AIDS Prevention and Treatment, The First Hospital of China Medical University, Shenyang, China

**Keywords:** Carbapenem-resistant *Klebsiella pneumoniae*, ST15, serum resistance, iron, virulence

## Abstract

ST15 has become one of the most common sequence types of carbapenem-resistant *Klebsiella pneumoniae* (CRKP) in China; however, the characteristics of ST15 CRKP remains elusive. This study included a total of 72 unique CRKP isolates, comprising ST15, ST11, ST307, and ST2237, collected from an intensive care unit between 2019 and 2022 to investigate the clinical and biological characteristics as well as the virulence of ST15 CRKP. Whole genome sequencing for 72 CRKP strains and transcriptome sequencing of ST11 and ST15 CRKP after serum exposure was also performed. The virulence of ST15 was similar to that of ST11-KL47, which carries the *rmpA2* and aerobactin virulence genes, and higher than that of ST11-KL10 and ST307. This may be attributed to its serum resistance. Whole-genome sequencing revealed that genes related to iron uptake (*sitA/C/D*), the type VI secretion system, and adherence were associated with the serum resistance of ST15. Furthermore, transcriptomic analysis showed that ST15 CRKP strains upregulated genes associated with iron transport and metabolism upon serum exposure. The effect of iron on serum resistance in ST15 was confirmed by supplementing with FeSO_4_ and FeCl_3_. Iron metabolism contributes to virulence by enhancing serum resistance in the emerging high-risk clone ST15 CRKP, with *sitA/C/D* potentially serving as key virulence genes.

## Introduction

Antimicrobial resistance poses a serious threat to global public health. In particular, carbapenem-resistant *Enterobacterales* (CRE) have attracted widespread attention and have been listed as a critical priority by the World Health Organization (WHO). The global distribution and prevalence of CRE have increased rapidly, especially those of carbapenem-resistant *Klebsiella pneumoniae* (CRKP). According to the WHO Global Antimicrobial Resistance and Use Surveillance System report 2022 (https://www.who.int/publications/i/item/9789240062702), the rate of bloodstream infections caused by CRKP increased from 11.5% in 2016 to 18.2% in 2020 worldwide. Additionally, nationwide surveillance in China revealed that the prevalence of CRKP increased from 7.6% in 2015 to 11.3% in 2021 (https://www.carss.cn/). Infections caused by CRE are typically associated with high mortality rates. A meta-analysis conducted in 2017 reported that the mortality rate for bloodstream infections caused by CRKP reached 54.3% [1].

Upon infection, bacteria are targeted by the body’s immune system, which determines the patient’s clinical manifestations and outcomes. The immune system, including neutrophils, monocytes, natural killer cells, and the complement system, plays an important role in eliminating the bacteria. However, bacteria can employ various virulent factors, such as pili, capsules, lipopolysaccharides (LPS), and iron carriers, to evade or counteract the immune response [2]. Previously, it was believed that resistance and virulence in *K. pneumoniae* did not overlap. However, hypervirulent CRKP (hv-CRKP), showing the convergence of carbapenem resistance and hypervirulence, has attracted global attention since it was first reported in 2018 [3,4].

CRKP strains from different countries and regions exhibit distinct molecular characteristics; therefore, epidemiological monitoring of these antimicrobial-resistant variants is highly important. The main sequence type (ST) of CRKP in China is ST11, with ST11-KL47 and ST11-KL64 being the most common sequence types. Recently, in China, ST11 has been found to be gradually shifting from ST11-KL47 to ST11-KL64; these two serotypes differ in terms of their biological characteristics and levels of virulence [5,6]. Meanwhile, the prevalence of other CRKP sequence types has also increased in recent years. For example, ST15 has been reported as predominant in several hospitals across multiple cities, becoming one of the most common CRKP sequence types in China [7–11]. Another study showed that ST15 CRKP has spread in localized hospitals and may be evolving toward hypervirulence; however, only 13 CRKP isolates were included in that study [12]. Moreover, mechanisms underlying its virulence characteristics have not yet been elucidated. In the present study, we investigated the biological and virulence characteristics of ST15 CRKP as well as the genetic mechanisms underlying this phenotype. The results provide insights that may aid in developing effective strategies for controlling CRKP infections.

## Materials and methods

### Participants and bacterial strains

This retrospective study was conducted at the First Hospital of China Medical University, a medical center located in northeastern China. Based on surveillance data, colonization or infection by *K. pneumoniae* was identified in 289 patients in the intensive care unit (ICU) between January 2019 and December 2022. Of these 289 patients, 102 were infected with CRKP isolates that exhibited resistance to imipenem (minimum inhibitory concentration [MIC] ≥ 4 μg/mL) or meropenem (MIC ≥4 μg/mL). A total of 72 unique isolates from 72 patients were available for further experiments.

Species identification was performed using VITEK 2 or MALDI-TOF MS (bioMérieux, France). Antimicrobial susceptibility testing for all isolates was conducted using the VITEK2 system, and the results were interpreted according to the criteria of the Clinical and Laboratory Standards Institute. Resistance to imipenem and meropenem was confirmed using the disc diffusion method or the microbroth dilution method [9]. Multilocus sequence typing (MLST) was performed to determine the sequence types of the CRKP isolates.

### Ethics statement

This study was approved by the Ethics Committee of the First Hospital of China Medical University (2024458). The ethics committee waived the requirement for informed consent for the following reasons: (1) the study was retrospective; (2) patient information was anonymized; and (3) the clinical isolates were collected and stored as part of routine diagnostic laboratory procedures, with no impact on patient care. The authors confirm that patient data confidentiality was maintained. This study adhered to the principles outlined in the Helsinki Declaration (https://www.wma.net/policies-post/wma-declaration-ofhelsinki/).

### Biofilm formation

Six strains randomly selected from ST11, ST15 and ST307 were subjected to biofilm formation assays, growth assays and serum killing assays. The biofilm formation assay was conducted using a previously described method, with some modifications [13]. Briefly, overnight cultures were adjusted to 10^7^ CFU/mL with fresh Luria – Bertani (LB) broth, and 200 µL aliquots were transferred to 96-well polystyrene plates. After incubation at 37°C for 24 h, the wells were gently washed and stained with 0.1% crystal violet. They were then washed three times, and 95% ethanol was added to solubilize the bound dye for OD_590_ measurements. Each assay was performed in triplicate on at least three separate occasions.

### Growth assay

Overnight cultures were adjusted to 10^6^ CFU/mL with fresh LB broth. LB broth without bacterial inoculation served as the negative control. OD_600_ was measured hourly for 12 h to generate growth curves.

### Serum killing assay

Serum resistance was assessed using a previously described procedure to evaluate *in vitro* virulence. Briefly, 25 µL exponential-phase bacterial culture (10^6^ CFU/mL) was mixed with 75 µL of pooled human serum obtained from 10 healthy individuals. The mixture was incubated at 37°C for 1, and 2 h, and viable bacteria were quantified by performing 10-fold serial dilutions and plating on Mueller-Hinton (MH) agar. Each experiment was repeated three times, and a representative result is presented. Additionally, bacterial survival after 2 h of serum incubation was evaluated for all 72 CRKP strains.

### Lactate dehydrogenase (LDH) assay

A549 cells were seeded into 96-well plates, and incubated at 37°C with different groups of CRKP strains at a multiplicity of infection (MOI) of 10. After 24 h, the supernatant was collected via centrifugation and transferred to a new 96-well plate. LDH working solution (Beyotime, China) was added, and the plate was incubated in the dark for 30 min. OD_490_ was then measured. Each experiment included a blank control and a negative control.

### Neutrophil killing

A total of 5 × 10^5^ neutrophils were incubated with different groups of CRKP strains (MOI = 10) at 37°C for 1 h. The cells were then lysed with Triton X-100 for 10 min and plated on MH agar using 10-fold serial dilutions. Colonies were counted the following day, and the survival index was calculated as follows: (colony count after neutrophil treatment/colony count without neutrophil treatment) × 100%. All experiments were performed in triplicate.

### Galleria mellonella infection assay

Based on a previous study, a *G. mellonella* infection model was established to evaluate *in vivo* virulence [14]. Briefly, bacterial cultures in the exponential growth phase were washed with sterile phosphate-buffered saline (PBS) and adjusted to 10^8^ CFU/mL. Ten *G. mellonella* larvae were then injected with 10 µL of the bacterial suspension and incubated at 37°C in the dark. Larvae injected with 10 µL of NTUH-K2044 and PBS served as positive and negative controls, respectively. Dead larvae were counted at specific time points over a 36-h period following injection.

### Experimental murine infection

4–6-week-old C57BL/6 mice were purchased from Liaoning Changsheng Biotechnology. The mice were randomly divided into five groups: (1) 21 mice inoculated with three ST11- KL47 strains (seven mice per strain); (2) 21 mice inoculated with three ST11- KL10 strains (seven mice per strain); (3) 21 mice inoculated with three ST15 strains (seven mice per strain); (4) 10 mice intraperitoneally injected with 10^6^ CFU of NTUH-K2044 as positive controls; and (5) 10 mice intraperitoneally injected with PBS as negative controls. For each test strain, 10^7^ CFU of CRKP was intraperitoneally inoculated into the mice. Mouse survival was monitored at designated time points, and survival curves were plotted. All animal experiments in this study were approved by the Department of Laboratory Animal Science of China Medical University (KT2024219) and conducted in accordance with the ARRIVE guidelines [15].

### Whole-genome sequencing and data analysis

DNA was extracted from 72 CRKP strains cultured overnight, and resequencing was performed using the Illumina NovaSeq platform. The Burrows-Wheeler Aligner program was used to align the filtered sequencing data to the reference genome. Additionally, three representative strains (ST11-KL47, ST11-KL10, and ST15-KL19) were selected for whole-genome sequencing using the PacBio long-read platform to obtain higher-quality draft genomes. PlasmidFinder (https://cge.cbs.dtu.dk//services/PlasmidFinder/) was used to analyze plasmid replicon and incompatible types, whereas oriTfinder (https://tool-mml.sjtu.edu.cn/oriTfinder/oriTfinder.html) was used to assess plasmid mobility and identify virulence and resistance genes. Pathogenwatch (https://pathogen.watch/) was used to determine virulence genes, resistance genes, STs, wzi types, and capsule (K) and outer LPS (O) types as well as to construct a midpoint-rooted phylogenetic tree using the phangorn package [16]. The phylogenetic tree was then visualized and embellished using EvolView (https://evolgenius.info//evolview-v2/). Finally, VFDB (http://www.mgc.ac.cn/VFs/) was used to further analyze virulence genes in the CRKP strains.

### Transcriptomic analysis

Three ST15 and three ST11 strains that were resistant and sensitive to serum killing, respectively, were incubated for 1 h with serum obtained from healthy volunteers. Untreated strains were used as controls. The strains were then washed twice with PBS, and RNA was extracted from the bacterial pellets using TRIzol, followed by DNase treatment. RNA sequencing was performed on the Illumina NovaSeq 6000 platform (Shanghai Lingen Biotechnology Co., Ltd.). The RPKM algorithm was used to analyze differentially expressed genes between groups. Genes with a fold change > 2 and false discovery rate (FDR) < 0.05 were considered differentially expressed. Gene Ontology (GO) functional enrichment and KEGG pathway enrichment analyses of the differentially expressed genes were performed online using the Lingbo MicroClass website (http://www.cloud.biomicroclass.com/).

### Statistical analysis

WHONET 5.6 was used for strain screening and antimicrobial resistance analysis, and SPSS 20.0 was used for statistical analysis. Quantitative data are expressed as mean ± standard deviation or median (interquartile range) and were compared using Student’s t-test or a nonparametric test, as appropriate. Qualitative data are presented as rates and were compared using chi-square test or Fisher’s exact test. A *p*-value < 0.05 was considered statistically significant. SAS was used to analyze the correlation between virulence genes and serum resistance, with *p* < 0.001 considered highly significant. Graphpad Prism 5.0 was used for data visualization.

## Results

### Clinical and biological characteristics of ST15 CRKP

**Figure 1.**
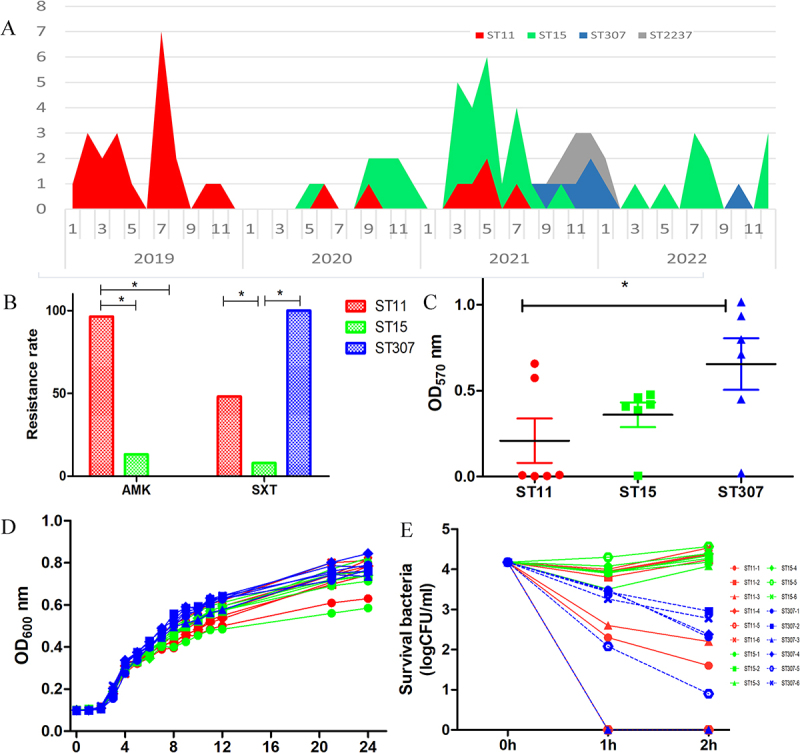
Sequence type conversion of CRKP isolates collected from the ICU and comparison of biological characteristics of ST15, ST11, and ST307 CRKP. (A) Distribution of CRKP sequence types over time; (B) resistance rates for three groups of CRKP to amikacin (AMK) and trimethoprim – sulfamethoxazole (SXT); biofilm formation (C), growth curves (D), and serum resistance (E) for three groups of CRKP.

Between January 2019 and December 2022, 72 CRKP strains from the ICU were recovered and subjected to MLST. The results showed that the predominant sequence type was ST15 (34 strains), followed by ST11 (27 strains), ST307 (6 strains), and ST2237 (5 strains). Notably, the distribution of CRKP sequence types changed over time (Figure 1(A)). ST11 CRKP was predominant in 2019, whereas ST15 became the predominant sequence type after 2020. ST307 emerged after September 2021; but did not cause an outbreak. The detailed characteristics of all 72 CRKP strains are provided in Table S1.

The ST15, ST11, and ST307 CRKP isolates differed significantly in their resistance rates to amikacin and trimethoprim – sulfamethoxazole (Figure 1(B)). Resistance rates to other antimicrobials did not show significant differences. Additionally, the biological characteristics of these isolates were analyzed using six randomly selected strains from each group. No significant differences were observed in growth curves or biofilm formation between ST11 and ST15 CRKP; however, ST307 CRKP exhibited higher levels of biofilm formation than ST11 (Figures 1(C,D)). Notably, the three CRKP sequence types displayed varying levels of resistance to serum killing (Figure 1(E)). All six ST15 CRKP isolates were resistant to serum killing, whereas all ST307 CRKP isolates were sensitive. In contrast, ST11 CRKP isolates exhibited various serum resistance, with some strains being resistant and others sensitive.

### Phylogenetic analysis and virulence genes

**Figure 2.**
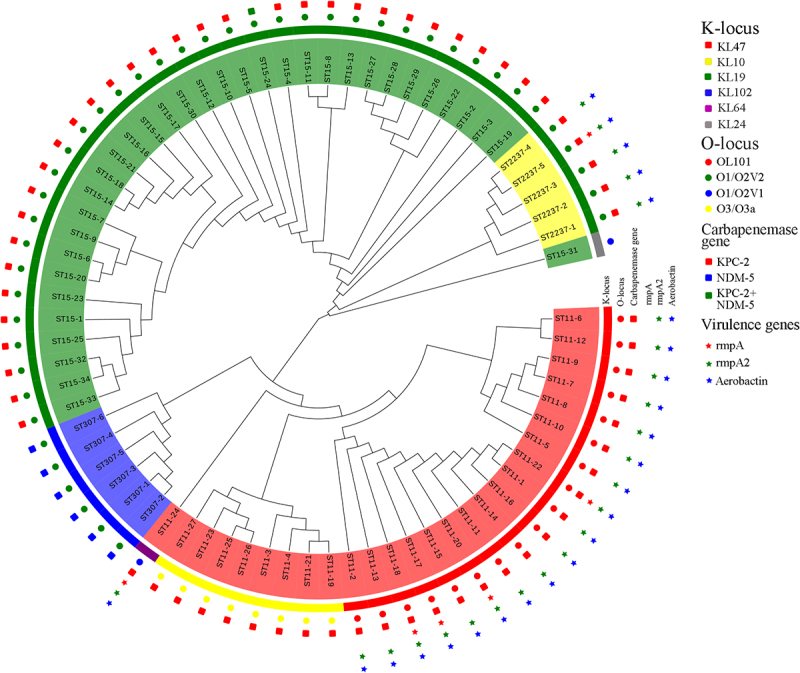
Phylogenetic analysis of the 72 CRKP isolates collected from the ICU. The outer rings show K genotype, O genotype, carbapenemase genes, and virulence genes.

**Figure 3.**
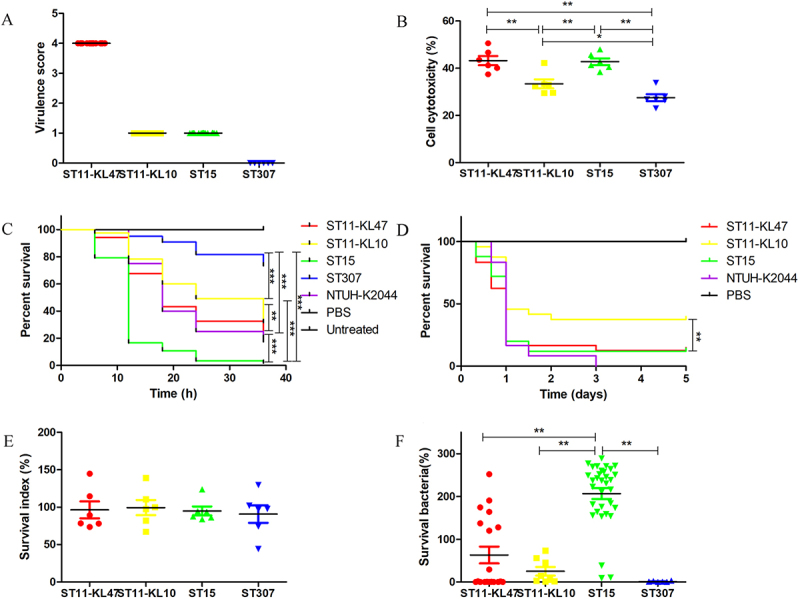
Characterization of the virulence traits of ST11-KL47, ST11-KL10, ST15, and ST307 CRKP. (A) Virulence scores; (B) cytotoxicity of CRKP on A549 cells detected by LDH assays; (C) survival curves of CRKP-infected *G. mellonella* larvae; (D) survival curves of CRKP-infected C57BL/6 mice; (E) percentage of surviving bacteria after incubation with neutrophils; (F) percentage of surviving bacteria after incubation with human serum.

Whole-genome sequencing was performed for all 72 CRKP isolates, and a phylogenetic tree was subsequently constructed (Figure 2). Regarding carbapenemase genes, all ST11 and ST15 isolates were positive for KPC-2, except for two strains: one that carried both KPC-2 and NDM-5 and another that was negative for any carbapenemase gene. All six ST307 isolates were positive for NDM-5. Moreover, the results showed that all ST15 isolates were positive for KL19, except for one. ST2237 was closely related to ST15. ST11 CRKP formed two distinct clusters: ST11-KL47 (18 strains) and ST11-KL10 (8 strains). ST15 and ST11-KL10 strains were negative for the *rmpA2* and aerobactin virulence genes, with a virulence score of 1. Conversely, ST11-KL47 and ST2237 carried these virulence genes and were classified as hv-CRKP, with a virulence score of 4. ST307 had a virulence score of 0 (Figure 3(A)).

Furthermore, *de novo* sequencing was performed for one representative stain each of ST15, ST11-KL47, and ST11-KL10. The results showed that the *rmpA2* and aerobactin virulence genes were located on the chromosome of ST11-KL47 rather than on a plasmid. Wang S. et al reported that virulence genes in ST11-KL47 transferred to chromosomes, which is consistent with our research, resulted in lower virulence and increased fitness [17]. Additionally, the plasmids carrying KPC-2 in ST15 and ST11-KL10 were predicted to have mobilizable potential, as indicated by oriTfinder [18]. Conversely, the KPC-2-carrying plasmid in ST11-KL47 was predicted to be non-mobilizable. The general features and resistance genes of the plasmids in ST15, ST11-KL47, and ST11-KL10 are summarized in Table S2.

### In vitro and in vivo experiments to evaluate the virulence of ST15 CRKP

An LDH assay was conducted to assess the *in vitro* virulence of ST15 CRKP. The results showed that ST15 CRKP exhibited virulence levels comparable to those of ST11-KL47 hv-CRKP. In contrast, ST11-KL10 and ST307 demonstrated lower virulence, with ST307 showing the lowest values (Figure 3(B)). Additionally, a *G. mellonella* infection model was used to evaluate the *in vivo* virulence of different CRKP groups. ST15 exhibited high virulence in the *G. mellonella* assay, followed by ST11, whereas ST307 displayed hypovirulence (Figure 3(C)). A mouse experiment was conducted to further compare the virulence of ST11 and ST15 CRKP strains. The results indicated that ST15 had virulence similar to ST11-KL47 and higher than that of ST11-KL10 (Figure 3(D)).

The resistance of different CRKP groups to neutrophil phagocytosis and serum killing was also assessed, as these are key mechanisms by which the human cellular and humoral immune systems combat bacterial infections. The four CRKP groups exhibited similar levels of resistance to neutrophil killing (Figure 3(E)). However, significant differences were observed in their resistance to serum killing. ST15 strains showed higher levels of serum resistance than ST11 and ST307 (Figure 3(F)). In summary, ST15 exhibited similar virulence to ST11-KL47 hv-CRKP, which may be attributed to enhanced resistance to serum killing.

### Genomic analysis of genes associated with serum resistance in ST15 CRKP

**Figure 4.**
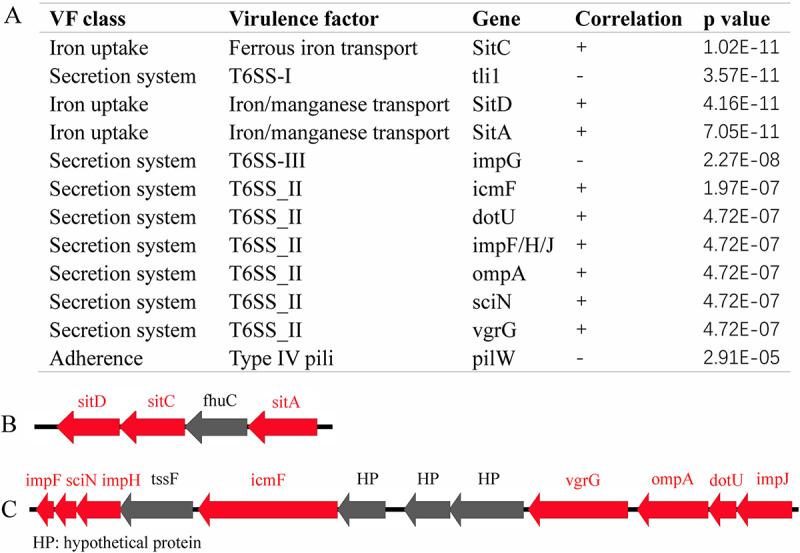
List of virulence genes associated with serum resistance in CRKP (A) and diagram of the structure of the *sitA/C/D* (B) and *T6SS_II* (C) gene clusters.

To identify genes associated with serum resistance in ST15, we analyzed the virulence genes of the 72 CRKP strains and examined their correlation with serum resistance. The results indicated that the virulence genes *sitC*, *tli1*, *sitD*, *sitA*, *impG*, *icmF*, and *dotU*, which are involved in functions such as iron uptake, type VI secretion system (T6SS), and adherence, were significantly associated with serum resistance (*p* < 0.001, Figure 4(A)). The gene structures of the *sitA/C/D* and *T6SS_II* clusters are shown in Figure 4(B,C), respectively.

### Transcriptomic analysis of ST15 CRKP after serum exposure

**Figure 5.**
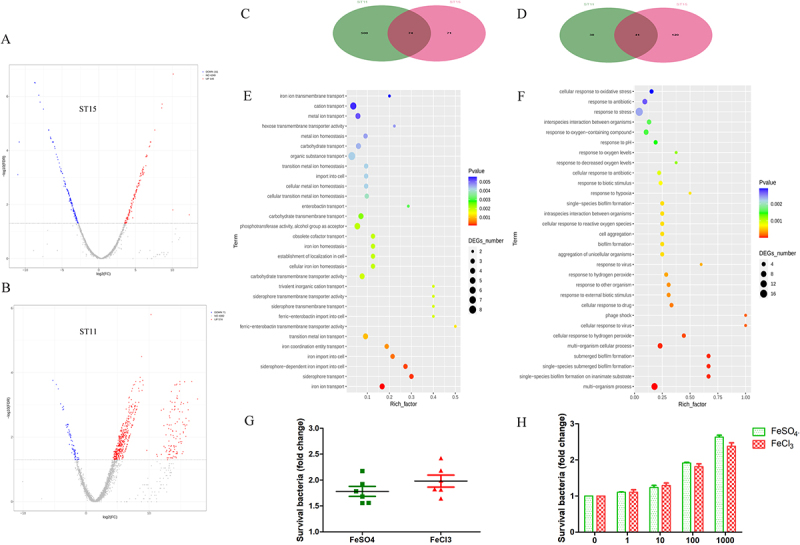
Transcriptomic analysis of ST15 and ST11 CRKP following serum exposure. Panels A and B show volcano plots of differentially expressed genes of ST15 (A) and ST11 (B) CRKP following incubation with human serum; panels C and D show Venn diagram illustrating the overlap and exclusivity of the upregulated genes (C) and downregulated genes (D) in ST15 and ST11 CRKP following serum exposure; panels E and F show GO enrichment analysis of upregulated genes (E) and downregulated genes (F) uniquely in ST15 CRKP; (G) effect of 100 mM FeSO_4_ and FeCl_3_ on the number of surviving bacteria following serum exposure; (H) effect of different concentrations of FeSO_4_ and FeCl_3_ on the number of surviving ST15 bacteria following serum exposure.

First, the upregulated and downregulated genes of ST15 and ST11 CRKP strains after serum exposure were analyzed. The results revealed that the serum killing-sensitive ST11 strains upregulated more genes than ST15 strains after incubating them with serum (Figure 5(A,B)). Venn diagrams illustrating the overlap and exclusivity of the upregulated and downregulated genes in ST15 and ST11 CRKP strains are shown in Figure 5(C,D), respectively. Additionally, GO enrichment analysis was performed for the genes uniquely upregulated and downregulated in ST15 CRKP (Figures 5(E,F)). It showed that the upregulated genes of ST15 CRKP were primarily enriched in functions related to iron transport and metabolism, whereas the downregulated genes were mainly associated with biofilm formation and cellular responses.

To further verify the effect of iron on serum killing, we evaluated the impact of FeSO_4_ and FeCl_3_ on the serum resistance exhibited by ST15. The number of surviving bacteria after the addition of 100 mM FeSO_4_ and FeCl_3_ was approximately twice that observed in the control group (Figure 5(G)). Moreover, the number of surviving bacteria following serum incubation increased in a concentration-dependent manner with both FeSO_4_ and FeCl_3_ (Figure 5(H)). Overall, these finding indicate that iron plays critical role in the serum resistance of ST15 and may be a major contributing factor. In particular, given that transcriptomic analysis showed that iron metabolism involved in serum resistance in ST15, it is speculated that *sitA/C/D* genes (associated with iron uptake) may serve as key virulence determinants contributing to the serum resistance of ST15.

## Discussion

ST15 CRKP exhibited similar virulence to ST11-KL47, which carries the *rmpA2* and aerobactin virulence genes, potentially due to its resistance to serum killing. Genomic analysis revealed that genes related to iron uptake, T6SS, and adherence may be associated with the serum resistance of ST15 CRKP. Transcriptomic analysis further confirmed that iron metabolism plays an important role in serum resistance. Based on the genomic and transcriptomic findings, it was concluded that iron metabolism contributes to virulence by enhancing serum resistance in ST15 CRKP, potentially through the involvement of *sitA/C/D*.

CRKP strains from different regions exhibit diverse molecular epidemiological characteristics, with distinct traits associated with virulence and transmission. In China, ST11 CRKP has been identified as the dominant sequence type. ST11 CRKP has evolved from KL47 to KL64 [19], with ST11-KL64 demonstrating enhanced virulence and transmissibility [20]. Patients with bloodstream infections caused by ST11-KL64 have shown significantly higher 30-day mortality (62.2%) than those infected with ST11-KL47 (52.8%). Recently, ST15 CRKP has been reported in multiple cities across China, emerging as the second most common sequence type [21,22], and has even replaced ST11 as the predominant CRKP in some hospitals. This indicates that ST15 CRKP possesses a strong transmission ability, second only to ST11 CRKP. A national multicenter study is needed to continuously monitor the epidemiological dynamics of ST15 CRKP. Moreover, the biological and virulence characteristics of ST15 CRKP remain to be fully elucidated.

In this study, ST15 CRKP demonstrated similar virulence to ST11-KL47 hv-CRKP carrying the *rmpA2* and aerobactin virulence genes and higher virulence than that of ST11-KL10 and ST307 CRKP. These findings are based on evidence obtained from *in vitro* and animal experiments; however, clinical validation is still needed. In recent years, cases of hv-CRKP have been increasingly reported not only in China, but also in India and other countries [23]. For example, a prospective cohort study conducted at a tertiary care hospital in South India reported an increased convergence of hypervirulence and carbapenem resistance in patients with CRKP bacteremia during 2021–2022 [24]. This indicates that hv-CRKP may pose a significant threat to public health in the future. Therefore, it is necessary to explore its pathogenic mechanisms to effectively combat hv-CRKP. ST15 CRKP strains were more resistant to serum killing than ST11 and ST307 strains but exhibited similar levels of resistance to neutrophil killing. This indicates that resistance to serum killing, rather than resistance to neutrophil phagocytosis, likely contributes to the high virulence of ST15 CRKP.

The complement system, a key contributor to serum resistance, is an essential component of the innate immune system and serves as the first line of defense against bacterial infections. It comprises over 30 plasma proteins, that promote inflammatory responses and facilitate bacterial killing through a cascade reaction involving three pathways: classical pathway, mannose-binding lectin pathway, and alternative pathway [25]. Conversely, bacteria can evade serum killing by employing various strategies, such as recruiting complement-activating proteins and inhibiting the activity of C3 and C5 convertases. This evasion is closely associated with bacterial structural components, including capsule polysaccharides, LPS, and outer membrane proteins [26]. For example, YadA, a transmembrane trimeric protein found on the surface of *Yersinia enterocolitica*, plays an important role in mediating bacterial binding to several complement components, such as the complement regulatory protein C4 and H factor [27]. The mechanism underlying serum resistance in *K. pneumoniae* remains unclear.

Genomic analysis was performed to identify the virulence determinants of ST15 CRKP and their association with serum resistance. The results showed that the genes *sitC*, *sitD*, *sitA*, *tli1*, and *impG* was associated with serum resistance. These genes are involved in functions such as iron uptake, T6SS, and adherence. Additionally, transcriptomic analysis of ST11 and ST15 CRKP following serum exposure revealed that the number of upregulated genes was significantly higher in ST11 than in ST15. Functional enrichment analysis of the upregulated genes in ST15 CRKP showed enrichment in pathways related to iron transport and metabolism, whereas the downregulated genes were associated with biofilm formation and cellular responses. Moreover, the addition of FeSO_4_ and FeCl_3_ promoted bacterial survival in serum in a concentration-dependent manner. Overall, genomic and transcriptomic analyses suggest that the serum resistance of ST15 CRKP is closely associated with iron metabolism.

Iron is involved in multiple fundamental physiological processes and is essential for the growth and survival of bacteria [28]. Additionally, iron metabolism plays an important role in bacterial resistance to serum killing. Iron has been shown to promote the growth of *Escherichia coli* and *Salmonella typhimurium* when exposed to serum [29]. *Vibrio vulnificus* strains carrying the siderophore-encoding gene *viuB* exhibit greater survival ability in serum than *viuB*-negative strains [30], indicating that iron is important for both serum survival and bacterial pathogenicity. This is consistent with our results, which suggest that iron metabolism is involved in the serum killing resistance of ST15 CRKP. Genomic analysis revealed that SitA/C/D proteins, known as Fur-regulated transporters of ferrous and manganese ions in Enterobacteriaceae, are associated with the serum resistance of ST15 CRKP. These findings support our hypothesis that *sitA/C/D* regulates iron metabolism, which in turn affects the serum resistance of ST15 CRKP. Specifically, a previous study demonstrated that *sitA* is associated with enhanced virulence of *K. pneumoniae* in mice [31], which aligns with our findings. Moreover, T6SS-positive *Acinetobacter baumannii* strains have been reported to show increased survival ability in human serum [32,33], which is also consistent with our findings.

In conclusion, the emerging high-risk clone ST15 CRKP exhibited similar virulence to ST11-KL47, which carries the *rmpA2* and aerobactin virulence genes, potentially due to its serum resistance. Genomic and transcriptomic analyses indicated that iron metabolism is associated with serum resistance in ST15 CRKP, with *sitA/C/D* likely serving as the virulence genes responsible for this function.

## Supplementary Material

Table S1.docx

Table S2.docx

## Data Availability

The raw sequence data reported in this paper have been deposited in National Center for Biotechnology Information that are publicly accessible at https://www.ncbi.nlm.nih.gov/sra/PRJNA1282784. Other data generated in this study are openly available in figshare at doi.org/10.6084/m9.figshare.28580882.
