# Supplementary material for: Iron metabolism contributes to virulence by enhancing serum resistance in the emerging high-risk clone ST15 CRKP
Source: Virulence. 2025 Oct 7;16(1):2569700. doi: 10.1080/21505594.2025.2569700 (PMC12505501; doi:10.1080/21505594.2025.2569700)
Supplement: Table S1.docx [file KVIR_A_2569700_SM7234.docx]

Table S1 Detailed characteristics of 72 CRKP strains from the ICU.

| ID | Specimen collection date | Specimen type | AMK | IPM | MEM | SXT | Carbapenemase genes | wzi | K-locus | O-locus | Virulence score | Resistance score | Aerobactin | rmpA2 | rmpA |
| --- | --- | --- | --- | --- | --- | --- | --- | --- | --- | --- | --- | --- | --- | --- | --- |
| ST11-1 | 2019/3/10 | sp | >=64 | >=16 | >=16 | >=16/304 | KPC-2 | 209 | KL47 | OL101 | 4 | 2 | ＋ | ＋ | - |
| ST11-2 | 2019/2/14 | sp | >=64 | >=16 | >=16 | <=1/19 | KPC-2 | 209 | KL47 | OL101 | 4 | 2 | ＋ | ＋ | - |
| ST11-3 | 2019/1/30 | sp | >=64 | >=16 | >=16 | >=16/304 | KPC-2 | 100 | KL10 | O3/O3a | 1 | 2 | - | - | - |
| ST11-4 | 2019/3/22 | sp | >=64 | >=16 | >=16 | >=16/304 | KPC-2 | 100 | KL10 | O3/O3a | 1 | 2 | - | - | - |
| ST11-5 | 2019/2/3 | bl | >=64 | >=16 | >=16 | >=16/304 | KPC-2 | 209 | KL47 | OL101 | 4 | 2 | ＋ | ＋ | - |
| ST11-6 | 2019/2/4 | ur | >=64 | >=16 | >=16 | >=16/304 | KPC-2 | 209 | KL47 | OL101 | 4 | 2 | ＋ | ＋ | - |
| ST11-7 | 2019/5/8 | sp | >=64 | >=16 | >=16 | >=16/304 | KPC-2 | 209 | KL47 | OL101 | 4 | 2 | ＋ | ＋ | - |
| ST11-8 | 2019/4/25 | sp | >=64 | >=16 | >=16 | >=16/304 | KPC-2 | 209 | KL47 | OL101 | 4 | 2 | ＋ | ＋ | - |
| ST11-9 | 2019/4/11 | bl | >=64 | >=16 | >=16 | >=16/304 | KPC-2 | 209 | KL47 | OL101 | 4 | 2 | ＋ | ＋ | - |
| ST11-10 | 2019/7/6 | sp | >=64 | >=16 | >=16 | >=16/304 | KPC-2 | 209 | KL47 | OL101 | 4 | 2 | ＋ | ＋ | - |
| ST11-11 | 2019/7/6 | bi | >=64 | >=16 | >=16 | <=1/19 | KPC-2 | 209 | KL47 | OL101 | 4 | 2 | ＋ | ＋ | - |
| ST11-12 | 2019/7/13 | se | >32 | >8 | >8 | >4/76 | KPC-2 | 209 | KL47 | OL101 | 4 | 2 | ＋ | ＋ | - |
| ST11-13 | 2019/7/15 | sp | >32 | >8 | >8 | <=1/19 | KPC-2 | 209 | KL47 | OL101 | 4 | 2 | ＋ | ＋ | - |
| ST11-14 | 2019/7/17 | sp | >=64 | >=16 | >=16 | <=1/19 | KPC-2 | 209 | KL47 | OL101 | 4 | 2 | ＋ | ＋ | - |
| ST11-15 | 2019/7/11 | sp | >32 | >8 | >8 | <=1/19 | KPC-2 | 209 | KL47 | OL101 | 4 | 2 | ＋ | ＋ | - |
| ST11-16 | 2019/7/31 | sp | 8 | >=16 | >=16 | <=1/19 | KPC-2 | 209 | KL47 | OL101 | 4 | 3 | ＋ | ＋ | - |
| ST11-17 | 2019/8/11 | sf | >=64 | >=16 | >=16 | <=1/19 | KPC-2 | 209 | KL47 | OL101 | 4 | 2 | ＋ | ＋ | ＋ |
| ST11-18 | 2019/8/17 | sp | >=64 | >=16 | >=16 | <=1/19 | KPC-2 | 209 | KL47 | OL101 | 4 | 2 | ＋ | ＋ | ＋ |
| ST11-19 | 2019/10/24 | sp | >=64 | >=16 | >=16 | >=16/304 | KPC-2 | 100 | KL10 | O3/O3a | 1 | 2 | - | - | - |
| ST11-20 | 2019/11/22 | ur | >=64 | >=16 | >=16 | <=1/19 | KPC-2 | 209 | KL47 | OL101 | 4 | 2 | ＋ | ＋ | ＋ |
| ST11-21 | 2020/6/28 | bl | >32 | >8 | >8 | >4/76 | KPC-2 | 100 | KL10 | O3/O3a | 1 | 2 | - | - | - |
| ST11-22 | 2020/9/14 | sp | >=64 | >=16 | >=16 | <=1/19 | KPC-2 | 209 | KL47 | OL101 | 4 | 2 | ＋ | ＋ | ＋ |
| ST11-23 | 2021/3/24 | ur | >=64 | >=16 | >=16 | <=1/19 | KPC-2 | 100 | KL10 | O3/O3a | 1 | 2 | - | - | - |
| ST11-24 | 2021/4/3 | sp | >=64 | >=16 | >=16 | >=16/304 | KPC-2 | 64 | KL64 | O1/O2v1 | 4 | 2 | ＋ | ＋ | ＋ |
| ST11-25 | 2021/5/15 | sp | >=64 | >=16 | >=16 | <=1/19 | KPC-2 | 100 | KL10 | O3/O3a | 1 | 2 | - | - | - |
| ST11-26 | 2021/5/16 | sp | >=64 | >=16 | >=16 | <=1/19 | KPC-2 | 100 | KL10 | O3/O3a | 1 | 2 | - | - | - |
| ST11-27 | 2021/7/19 | sp | >=64 | >=16 | >=16 | <=1/19 | KPC-2 | 100 | KL10 | O3/O3a | 1 | 2 | - | - | - |
| ST15-1 | 2020/5/28 | ur | 4 | >=16 | >=16 | <=1/19 | KPC-2 | 19 | KL19 | O1/O2v2 | 1 | 2 | - | - | - |
| ST15-2 | 2020/9/28 | sp | 4 | >=16 | >=16 | <=1/19 | KPC-2 | 19 | KL19 | O1/O2v2 | 1 | 2 | - | - | - |
| ST15-3 | 2020/10/11 | bl | <=2 | >=16 | >=16 | <=1/19 | KPC-2 | 19 | KL19 | O1/O2v2 | 1 | 2 | - | - | - |
| ST15-4 | 2020/10/27 | ur | 8 | >=16 | >=16 | <=1/19 | KPC-2 | 19 | KL19 | O1/O2v2 | 1 | 2 | - | - | - |
| ST15-5 | 2020/11/15 | ur | 8 | 8 | >=16 | <=1/19 | KPC-2 | 19 | KL19 | O1/O2v2 | 1 | 2 | - | - | - |
| ST15-6 | 2020/11/27 | sp | 4 | >=16 | >=16 | <=1/19 | KPC-2 | 19 | KL19 | O1/O2v2 | 1 | 2 | - | - | - |
| ST15-7 | 2020/12/30 | bl | 16 | >=16 | >=16 | <=1/19 | KPC-2 | 19 | KL19 | O1/O2v2 | 1 | 2 | - | - | - |
| ST15-8 | 2021/3/14 | sp | <=8 | >8 | >8 | <=1/19 | KPC-2 | 19 | KL19 | O1/O2v2 | 1 | 2 | - | - | - |
| ST15-9 | 2021/3/17 | bl | 4 | >=16 | >=16 | 4/76 | KPC-2 | 19 | KL19 | O1/O2v2 | 1 | 2 | - | - | - |
| ST15-10 | 2021/3/19 | ur | 4 | 8 | >=16 | <=1/19 | KPC-2 | 19 | KL19 | O1/O2v2 | 1 | 2 | - | - | - |
| ST15-11 | 2021/3/31 | sp | 4 | 8 | >=16 | <=1/19 | KPC-2 | 19 | KL19 | O1/O2v2 | 1 | 2 | - | - | - |
| ST15-12 | 2021/4/5 | sp | 4 | >=16 | >=16 | <=1/19 | KPC-2 | 19 | KL19 | O1/O2v2 | 1 | 2 | - | - | - |
| ST15-13 | 2021/4/9 | sp | 4 | >=16 | >=16 | <=1/19 | KPC-2 | 19 | KL19 | O1/O2v2 | 1 | 2 | - | - | - |
| ST15-14 | 2021/4/12 | sp | <=2 | 4 | >=16 | <=1/19 | KPC-2 | 19 | KL19 | O1/O2v2 | 1 | 2 | - | - | - |
| ST15-15 | 2021/5/2 | fl | <=2 | >=16 | >=16 | 8/152 | KPC-2 | 19 | KL19 | O1/O2v2 | 1 | 2 | - | - | - |
| ST15-16 | 2021/5/12 | sp | <=2 | >=16 | >=16 | 2/38 | KPC-2 | 19 | KL19 | O1/O2v2 | 1 | 2 | - | - | - |
| ST15-17 | 2021/5/15 | sp | <=2 | >=16 | >=16 | <=1/19 | KPC-2 | 19 | KL19 | O1/O2v2 | 1 | 2 | - | - | - |
| ST15-18 | 2021/5/30 | sp | <=2 | >=16 | >=16 | <=1/19 | KPC-2 | 19 | KL19 | O1/O2v2 | 1 | 2 | - | - | - |
| ST15-19 | 2021/6/17 | ca | 4 | >=16 | >=16 | <=1/19 | KPC-2 | 19 | KL19 | O1/O2v2 | 1 | 2 | - | - | - |
| ST15-20 | 2021/7/4 | fl | 8 | >=16 | >=16 | 2/38 | KPC-2 | 19 | KL19 | O1/O2v2 | 1 | 2 | - | - | - |
| ST15-21 | 2021/7/15 | fl | 4 | >=16 | >=16 | <=1/19 | KPC-2 | 19 | KL19 | O1/O2v2 | 1 | 2 | - | - | - |
| ST15-22 | 2021/7/31 | sp | 4 | >=16 | >=16 | <=1/19 | KPC-2 | 19 | KL19 | O1/O2v2 | 1 | 2 | - | - | - |
| ST15-23 | 2021/8/3 | ur | 8 | >=16 | >=16 | <=1/19 | KPC-2 | 19 | KL19 | O1/O2v2 | 1 | 2 | - | - | - |
| ST15-24 | 2021/10/28 | fl | 4 | >=16 | >=16 | <=1/19 | KPC-2, NDM-5 | 19 | KL19 | O1/O2v2 | 1 | 2 | - | - | - |
| ST15-25 | 2022/3/22 | fl | 4 | >=16 | >=16 | <=1/19 | KPC-2 | 19 | KL19 | O1/O2v2 | 1 | 2 | - | - | - |
| ST15-26 | 2022/5/24 | sp | <=2 | >=16 | >=16 | <=1/19 | KPC-2 | 19 | KL19 | O1/O2v2 | 1 | 2 | - | - | - |
| ST15-27 | 2022/7/8 | bl | 4 | >=16 | >=16 | <=1/19 | KPC-2 | 19 | KL19 | O1/O2v2 | 1 | 2 | - | - | - |
| ST15-28 | 2022/7/29 | sp | <=2 | 8 | >=16 | <=1/19 | KPC-2 | 19 | KL19 | O1/O2v2 | 1 | 2 | - | - | - |
| ST15-29 | 2022/7/28 | sp | 4 | 8 | >=16 | <=1/19 | KPC-2 | 19 | KL19 | O1/O2v2 | 1 | 2 | - | - | - |
| ST15-30 | 2022/8/1 | bl | <=2 | >=16 | >=16 | 2/38 | KPC-2 | 19 | KL19 | O1/O2v2 | 1 | 2 | - | - | - |
| ST15-31 | 2022/8/6 | fl | >=64 | >=16 | >=16 | 4/76 | Neg | 24 | KL24 | O1/O2v1 | 0 | 0 | - | - | - |
| ST15-32 | 2022/12/23 | sp | 4 | 8 | >=16 | 2/38 | KPC-2 | 19 | KL19 | O1/O2v2 | 1 | 2 | - | - | - |
| ST15-33 | 2022/12/24 | fl | <=2 | >=16 | >=16 | <=1/19 | KPC-2 | 19 | KL19 | O1/O2v2 | 1 | 2 | - | - | - |
| ST15-34 | 2022/12/22 | sp | 4 | 8 | >=16 | 2/38 | KPC-2 | 19 | KL19 | O1/O2v2 | 1 | 2 | - | - | - |
| ST2237-1 | 2021/10/10 | bl | >=64 | >=16 | 4 | <=1/19 | KPC-2 | 19 | KL19 | O1/O2v2 | 4 | 2 | 1 | 1 | ＋ |
| ST2237-2 | 2021/11/17 | sp | >=64 | 8 | >=16 | 2/38 | KPC-2 | 19 | KL19 | O1/O2v2 | 4 | 2 | 1 | 1 | ＋ |
| ST2237-3 | 2021/11/22 | fl | >=64 | 8? | 4 | <=1/19 | KPC-2 | 19 | KL19 | O1/O2v2 | 4 | 2 | 1 | 1 | ＋ |
| ST2237-4 | 2021/12/14 | fl | >=64 | >=16 | 4 | <=1/19 | KPC-2 | 19 | KL19 | O1/O2v2 | 4 | 2 | 1 | 1 | ＋ |
| ST2237-5 | 2022/1/15 | sp | >=64 | 8 | 4 | <=1/19 | KPC-2 | 19 | KL19 | O1/O2v2 | 4 | 3 | 1 | 1 | ＋ |
| ST307-1 | 2021/9/22 | ur | <=2 | >=16 | >=16 | >16/304 | NDM-5 | 173 | KL102 | O1/O2v2 | 0 | 2 | - | - | - |
| ST307-2 | 2021/11/15 | ur | 16 | >=16 | >=16 | >16/304 | NDM-5 | 173 | KL102 | O1/O2v2 | 0 | 2 | - | - | - |
| ST307-3 | 2021/12/13 | fl | 16 | >=16 | >=16 | >16/304 | NDM-5 | 173 | KL102 | O1/O2v2 | 0 | 2 | - | - | - |
| ST307-4 | 2021/12/18 | ur | <=2 | >=16 | 8 | >16/304 | NDM-5 | 173 | KL102 | O1/O2v2 | 0 | 2 | - | - | - |
| ST307-5 | 2022/1/4 | bi | 4 | >=16 | >=16 | >16/304 | NDM-5 | 173 | KL102 | O1/O2v2 | 0 | 2 | - | - | - |
| ST307-6 | 2022/10/8 | fl | <=2 | >=16 | >=16 | >16/304 | NDM-5 | 173 | KL102 | O1/O2v2 | 0 | 2 | - | - | - |

sp: sputum; bl: blood; ur: urine; bi: bile; se: secretion; sf: cerebrospinal fluid; fl: fluid; ca: catheter. AMK: amikacin; IPM: imipenem; MEM: meropenem; SXT: sulfamethoxazole/trimethoprim.
