# Supplementary material for: Iron metabolism contributes to virulence by enhancing serum resistance in the emerging high-risk clone ST15 CRKP
Source: Virulence. 2025 Oct 7;16(1):2569700. doi: 10.1080/21505594.2025.2569700 (PMC12505501; doi:10.1080/21505594.2025.2569700)
Supplement: Table S2.docx [file KVIR_A_2569700_SM7233.docx]

Table S2 General features and resistance genes of plasmids in ST11-KL47, ST11-KL10 and ST15.

| Characteristics | ST11-KL47 | ST11-KL10 | | ST15 | |
| --- | --- | --- | --- | --- | --- |
|  | plasmid 1 | plasmid 1 | plasmid 2 | plasmid 1 | plasmid 2 |
| Length | 92761 | 221374 | 145659 | 231195 | 136725 |
| GC content(%) | 55.55 | 51.50 | 54.39 | 52.60 | 52.58 |
| No. of ORF | 4 | 31 | 14 | 43 | 62 |
| Incapability group | lncFII | IncFIB(K) | IncFII | IncFIB(K) | lncFIIK |
| Mobial ability | No | No | Yes | Yes | Yes |
| OriT(start…stop)(bp) | / | / | 110803..110888 (-) | 21390..21439 (-) | 22905..22961 (-) |
| Relaxase(start…stop)(bp) | / | / | / | 050498..51007 (+) | 050924..55066 (+) |
| T4CP(start…stop)(bp) | / | 0116542..116790 (-) | / | [48191..50092](http://tool-mml.sjtu.edu.cn/oriTfinder/report_conjugal.php?job_id=puoJxPI4y#T4CP) (+) | 48615..50924 (+) |
| T4SS(start…stop)(bp) | / | 118602..143720 | 110236..121629 | [20832..56517](http://tool-mml.sjtu.edu.cn/oriTfinder/report_conjugal.php?job_id=puoJxPI4y#T4SS)/222721..228732 | 22353..56950/113978..136724 |
| Resistance genes | blaKPC-2  blaSHV-12 | aac(3)-IId, blaLAP-2, qnrS1, tetA, tetD, blaTEM-1 | blaKPC-2,  blaTEM-1, blaSHV-12 | blaTEM-1, blaCTX-M-15, blaOXA-1, acc(6')-Ib, tetA | blaKPC-2 |
| Virulence factors | / | / | / | / | / |

ORF: open reading frame; OriT: origin of transfer; T4CP: type IV coupling proteins; T4SS: type IV secretion system.
